# pH-Sensitive Targeting of Tumors with Chemotherapy-Laden Nanoparticles: Progress and Challenges

**DOI:** 10.3390/pharmaceutics14112427

**Published:** 2022-11-10

**Authors:** Zuha Imtiyaz, Jiaxi He, Qixin Leng, Atul K. Agrawal, A. James Mixson

**Affiliations:** 1 Department of Pathology, University Maryland School of Medicine, 10 S. Pine St., University of Maryland, Baltimore, MD 21201, USA; 2 20511 Seneca Meadows Pkwy, Suite 260, RNAimmune, Germantown, MD 20876, USA

**Keywords:** polymers, histidine, imidazole, doxorubicin, nanoparticles, micelles, tumor pH

## Abstract

Accumulating chemotherapeutic drugs such as doxorubicin within a tumor while limiting the drug dose to normal tissues is a central goal of drug delivery with nanoparticles. Liposomal products such as Doxil^®^ represent one of the marked successes of nanoparticle-based strategies. To replicate this success for cancer treatment, many approaches with nanoparticles are being explored in order to direct and release chemotherapeutic agents to achieve higher accumulation in tumors. A promising approach has been stimulus-based therapy, such as the release of chemotherapeutic agents from the nanoparticles in the acidic environments of the tumor matrix or the tumor endosomes. Upon reaching the acidic environments of the tumor, the particles, which are made up of pH-dependent polymers, become charged and release the entrapped chemotherapy agents. This review discusses recent advances in and prospects for pH-dependent histidine-based nanoparticles that deliver chemotherapeutic agents to tumors. The strategies used by investigators include an array of histidine-containing peptides and polymers which form micelles, mixed micelles, nanovesicles, polyplexes, and coat particles. To date, several promising histidine-based nanoparticles have been demonstrated to produce marked inhibition of tumor growth, but challenges remain for successful outcomes in clinical trials. The lessons learned from these histidine-containing particles will provide insight in the development of improved pH-dependent polymeric delivery systems for chemotherapy.

## 1. Introduction

Targeting of chemotherapy is necessary to avoid the toxic effects of the free drugs. Doxil^®^, which incorporates doxorubicin (Dox) within liposomes, was developed to avoid such undesirable side effects as cardiotoxicity [1,2]. Accumulation of the drug within the tumor while limiting the Dox dose to the heart partially explains the drug’s reduced toxicity. Another factor in the clinical utility of Doxil^®^ (and other liposome–Dox preparations) is that high levels of Dox can be loaded into the liposomes. This is based on the clever strategy of loading the weakly basic Dox into the acidic interior of the liposomes [3]. Today, improved methods and carriers are still being explored to deliver Dox. Unfortunately, other hydrophobic chemotherapy agents, such as paclitaxel (PTX), have not been effectively incorporated into liposomes [4]. Like Dox, these chemotherapy drugs have serious side effects, and targeted therapy would likely increase the therapeutic window of these drugs. As a result, the development of carriers of these hydrophobic drugs is an ongoing area of investigation.

Using pH-sensitive polymers to make up nanoparticles is an attractive approach for delivering chemotherapy agents selectively to acidic environments such as the tumor matrix and endosomes [5]. These polymers contain weakly acidic or basic groups and have been exploited as pH-responsive polymers, inspiring advances in drug delivery systems. These include polybasic polymers such as poly(2-(diethylamino)ethyl methacrylate) (PDEAEMA) [6,7], poly(2-(dipropylamino)ethyl methacrylate (PDPA or PDPAEMA) [8], poly(beta-amino ester) PBAE [9], and poly-L-histidine (PLH) [10], and polyacidic polymers such as polyacrylic acid [11]. To take advantage of the low pH surrounding tumors and in tumor endosomes [12,13], the pKa of the polymers making up the micelles or nanoparticles is usually between pH 5 and 7.5. When the pKa of the polymeric molecule is close to the pH of the surrounding milieu, the polymers become protonated within the particle, leading to charge–charge repulsion or phase transition (e.g., hydrophobic to hydrophilic) [14]. This disruption of nanoparticles within the acidic environment may occur extracellularly or intracellularly. Both these sites, which have different ranges of acidic pH, have been used to deliver hydrophobic drugs specifically to tumors.

Nanoparticles and the drugs they carry are exposed to dramatically different pH ranges once they leave the blood vessels that feed the tumor [15]. While the extracellular environment and the endosomes are acidic, the intracellular pH of a tumor cell is alkaline. The extracellular acidic environment of a tumor (T_E_) offers the potential to target the tumor. There are relatively few other tissues (e.g., renal proximal tubule, gastric lumen) or diseases (e.g., arthritic joints) that have an acidic environment [16,17,18,19] The acidity of the T_E_ ranges from a pH of 6.5 to 7.2, and several mechanisms contribute to this acidity. Although this extracellular tumor acidity, first noted by Warburg, was initially thought to be due to enhanced glycolysis (aerobic and anaerobic) and increased levels of lactic acid [12,20], the reason for the acidity is still being examined. Increased glycolytic pathway activity is intrinsic to almost all cancer cells, whether they are located near or far from the blood vessels. Because of activating mutations, some cancers have a higher rate of glycolysis than others and this can affect the T_E_. Interestingly, due to increased glycolysis and enhanced glucose transport, the administration of glucose may further lower the extracellular pH of a tumor [21,22,23]. In addition to the production of lactic acid, the pentose pathway, sodium–hydrogen exchanges (particularly NHE1), and carbonic acid anhydrase activity may have important roles lowering the extracellular pH [24,25,26].

Because of the increased acidity of tumors, a pH gradient exists between normal tissues and perivascular tumor cells, enabling targeting of the tumor by pH-sensitive nanoparticles. Within a tumor, however, spatial–temporal differences in the extracellular pH of the tumor exist and these differences are dependent on the size of the tumor, the blood vessel density, the quality and organization of the blood vessels, and the lymphatics [27,28]. While the extracellular pH in necrotic areas of tumors has been studiously avoided in many studies, the pH of hypoxic (non-necrotic) regions has been found to be more acidic than that of regions adjacent to the vessels, primarily because of the increased production of CO_2_ and protons (lactic acid) associated with poor perfusion [29]. 

Although some pH-dependent nanoparticles have been designed to disintegrate in the extracellular environment, many particles remain partially intact. With their increased charge, these particles likely have enhanced absorption onto the negatively charged surface of tumor cells, resulting in greater endosomal uptake. After the uptake of the particles into the cell’s endosomes, the vesicles become progressively more acidic, with late endosomes reaching a pH of 5. With this lower pH, the likelihood that the pH-dependent particles will be disrupted (i.e., charge–charge repulsion) increases, resulting in the release of the hydrophobic drug. The second function that pH-dependent polymers may have in drug release is the disruption of endosomes. Two mechanisms have been proffered, and they are not mutually exclusive. Upon protonation of pH-dependent polymers (and nanoparticles), endosomes burst through osmotic swelling or by direct interaction of the endosomal membrane with the charged polymers [30,31,32]. 

This review focuses on pH-dependent histidine-based nanoparticles that deliver chemotherapeutic agents to tumors. These pH-dependent drug-laden nanoparticles were one of the earliest and most widely reported to be disrupted in the acidic environments of the tumor. Their early use in chemotherapy was undoubtedly due to their safety profile and the properties of imidazole–histidine polymers in the delivery of nucleic acids [14,33,34,35]. The nitrogen atom on an imidazole ring with unpaired electrons can attract protons between pH 5 and 7 and buffer acidic environments such as those in endosomes, resulting in the lysis of endosomes. It is essential that endosomes are lysed for nucleic acids to be released into the cytosol. Without this release, the endosomes will merge with the lysosomes and the nucleic acids will be degraded. Similarly, endosomal lysis, as well as buffering the acidity of endosomes, may aid in the release of hydrophobic drugs, particularly drugs such as the amino-containing Dox. Since endosomal lysis is relatively rare inside cells [36,37], buffering endosomes with histidine-containing nanoparticles may play a large role in the transport of Dox into the cytosol. Nevertheless, it has been suggested that small molecules (e.g., drugs) escape more commonly than higher-molecular-weight nucleic acids, probably because of minor breaks in the endosomal membrane [37]. Moreover, in contrast to the release of nucleic acids extracellularly, whereupon rapid enzymatic degradation occurs, the release of chemotherapeutic agents such as camptothecin analogs into the acidic T_E_ may enhance their antitumor efficacy [38]. 

To cover the wide range of designs of histidine-rich polymers and therapeutic agents incorporated into nanoparticles to target tumors, we have divided this review into four sections based on the polymer and nanoparticle composition: (1) linear imidazole-modified peptides and polymers; (2) branched copolymers; (3) dual delivery of nucleic acids and chemotherapy agents; and (4) composites. While linear and branched copolymers form nanoparticles that incorporate a hydrophobic chemotherapeutic agent, dual-delivery nanoparticles have a cationic and hydrophobic layer to bind to the nucleic acid and the drug, respectively. The nanoparticles formed of polymers and copolymers include micelles, mixed micelles, non-micelle nanoparticles such as nanovesicles, lipopolyplexes, and polyplexes. Moreover, diverse methods have been used to create these particles, such as direct dissolution, diafiltration, dialysis, thin-film hydration with and without dialysis, emulsion with solvent evaporation, and microfluidics. Of these methods, thin-film hydration with dialysis is the most commonly used to prepare histidine-containing nanoparticles.

## 2. Linear Imidazole-Modified Peptides and Polymers

Using block copolymers with a poly-L-histidine (PLH) domain, the Bae group conducted a series of seminal studies on the release of hydrophobic drugs from micelles in the acidic extracellular environment of tumors [10,39,40,41]. After comparing different molecular weights of the PLH segments (prepared by ring-opening polymerization), the molecular weight of 5000 was selected for the PLH component of the copolymer because of the lower critical micelle concentration (CMC) (2.3 μg/mL). Interestingly, the addition of polyethylene glycol (PEG) to the PLH domain (PEG-PLH) increased the pKa from 6.5 to 7.0. As with most of the histidine-containing micelles discussed in this review, the CMC of the PEG-PLH micelles was inversely correlated with the pH. The pH dependence of the CMC and the transmittance of PEG-PLH micelles were consistent with the protonation of the imidazole groups and their disruption at mildly acidic pH levels. Although many pH-buffering micelles increased in size at acidic pH levels, these micelles became smaller as the pH was lowered [10]. The PEG-PLH micelles released about 42%, 75%, and 85% of the Dox at pH 7.4, 6.8, and 5.0, respectively, over twenty-four hours at 37 °C [39]. The enhanced release of Dox from micelles at lower pH levels was further corroborated by the increased inhibition of MCF-7 cancer cells in more acidic media [39,40]. The group also determined that Dox-loaded PEG-PLH micelles had improved pharmacokinetics, enhanced tumor accumulation, and reduced tumor size compared to free Dox [40]. 

To improve the stability at physiological pH and the release of Dox at the mildly acidic pH levels found in the extracellular tumor environment, a blend of PEG-PLH and PEG- poly-L-lactide (PEG-PLA) micelles was investigated [39]. Compared to other PEG-PLH/PEG-PLA mixed micelles, the blend of PEG-PLH (75%) and PEG-PLA (25%) micelles showed improved release profiles for Dox at mildly acidic pH levels, reflective of the extracellular pH of tumors. While about 30% of the Dox was released from the optimal mixed micelle preparation at pH 7.4, nearly 75% was released at pH 6.8 over twenty-four hours. Concomitant with the release kinetics, the 75:25 mixed micelles showed enhanced cytotoxic activity toward MCF-7 cells incubated in media at pH 6.8 [39]. Furthermore, when Dox-loaded PEG-PLA/PEG-PLH micelles were decorated with the folate ligand, their inhibition of MCF-7 and drug-resistant MCF-7-Dox^R^ cells was significantly greater than that of the untargeted micelles (Table 1). Notably, free Dox had little effect on the MCF-7-Dox^R^ xenografts. In contrast, the Dox-loaded pH-sensitive micelles, particularly the folate-targeted micelles, had a marked effect on the growth of the xenografts [41]. In a later report, the authors indicated that long-term stability was an issue for these micelles [42]. 

**Figure 1:**
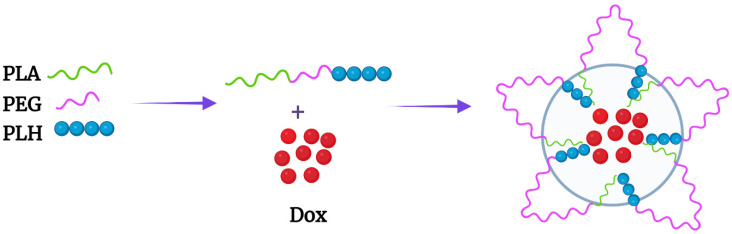
Flower-like micelle formed by the PLA-b-PEG-b-PLH triblock copolymer. Dox was incorporated within the PLA and PLH hydrophobic core.

**Figure 2:**
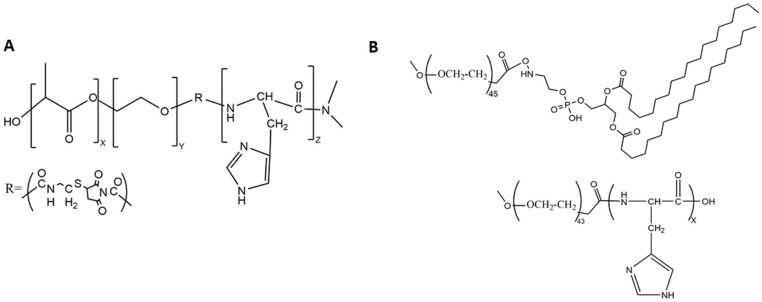
Chemical structures of the (**A**) triblock copolymer and (**B**) the block copolymers mPEG-PLH and PEG-DSPE, which formed micelles. The numbers of monomeric units in the block copolymer are represented by X, Y, and Z.

The improved kinetics of Dox release from mixed micelles with the hydrophobic poly-L-lactide (PLA) copolymer has stimulated interesting designs with triblock PLH-containing copolymers [43,44]. The Bae group designed an interesting PLA-PEG-PLH triblock copolymer which self-assembled into flower-like micelles (Figure 1 and Figure 2A). While the sandwich hydrophilic PEG segment was on the surface, the PLA and PLH segments on the ends made up the inner core. These micelles were approximately 80 nm in size at pH 7.4 and swelled to 580 nm at pH 6.6 [43]. Related to these size changes, the cumulative release of Dox from the micelles was 35% higher at pH 6.8 than the release at pH 7.4. The amounts of Dox released at various pH levels and the in vitro antitumor efficacy of these triblock micelles were similar to those of the mixed micelles [39].

Liu et al. reported somewhat different findings for micelles prepared with the same components but in a different order, a triblock mPEG-b-PLH-PLA copolymer [44]. In contrast to the PEG being sandwiched between two hydrophobic polymers, PEG was external to these domains. In a twenty-four hour period, about 35% and 80% of the Dox was released from the micelles at pH 7.4 and pH 5.0, respectively. Because there was no quick release of Dox from these micelles at pH 5, the authors speculated that Dox was primarily located in the hydrophobic PLA core at this acidic pH. The greater degree of polymerization of PLA compared to other block polymers [43,45] may have played a role in the entrapment of Dox at the lower pH. Significant amounts of Dox were unlikely to have been released from these micelles at pH values between 6.3 and 7.0 since no burst release was observed at pH 5.0. In any event, no release data were reported at a pH of 6.3, even though the micelles reached their maximum size at this pH prior to their becoming smaller. In contrast, there was a burst release of Dox at pH 6.5 and 5.0 in which PLH formed the inner core of the micelle (mPEG-PLA-PLH) [46]. Therefore, the order of the triblock polymer and perhaps the length of the polymeric block components may be important in determining whether micelles release Dox at mildly acidic pH levels between 6.5 and 7.0.

Compared to PLA, the copolymer poly(lactide-co-glycolide) (PLGA) is generally preferred because its biocompatibility, biodegradability, and mechanical strength can be controlled by varying the ratios of its monomers. Li et al. synthesized block copolymers of PLGA and tocopheryl polyethylene glycol succinate (TPGS-PLGA) with or without poly-L-histidine (TPGS-PLGA-PLH) [45]. Among these components, the PEG-containing TPGS segment formed the outer shell, improving the stability of the nanoparticles and inhibiting the multidrug resistance (MDR) transporter, while both the hydrophobic PLGA middle and PLH inner shell components entrapped Dox efficiently. Moreover, the Dox-loaded particles with PLH showed enhanced release of Dox at acidic pH and more significant cytotoxicity toward Dox-sensitive and -resistant breast cancer cells compared to particles without histidine. Although these results demonstrated the importance of PLH in the release of Dox [45], the Dox readily leaked from the cores of the two nanoparticles at pH 7.4 (TPGS-PLGA-PLH, TPGS-PLGA, ~55% in twelve hours)**.**

To reduce the release of Dox from nanoparticles, Johnson et al. synthesized a diblock copolymer composed of poly(2-hydroxyethyl methacrylate (pHEMA) and PLH domains (p(HEMA)-b-PLH) [47]. The number of monomeric histidines in the PLH domain markedly affected the biophysical characteristics of the micelle and the release profile of the Dox. The pHEMA component formed a hydrophilic shield, whereas the polyhistidine formed a hydrophobic core incorporating the Dox. Upon varying the number of histidines (15, 25, 35, and 45) in the PLH domain, the size of the Dox-loaded micelle was affected, ranging in size from about 124 to 194 nm. The more histidines in the diblock copolymer, the larger the Dox-loaded micelle and the greater the release rate of Dox at physiological pH and acidic pH. Despite the differences in size and release rates of Dox at different pH levels, the micelles with varying histidine content showed similar cytotoxicity for cancer cells yet reduced cytotoxicity compared to free Dox. Notably, cytotoxicity was progressively increased when the Dox-loaded micelles were incubated with the cells at lower pH levels. In a later study from the same group, a similar pH-dependent enhanced release of Dox was observed from micelles formed from the triblock copolymer (PEG- p(Lys)_25_-p(His)_100_) [48,49]. Although nucleic acids could presumably have been loaded into these micelles, only Dox was. It is possible that the nearly 50% release of Dox from the micelles at pH 7.4 was due to the self-repelling poly-L-lysine layer, and, with the addition of siRNA to neutralize the poly-L-lysine component, the release of the drug might have been reduced, as reported by others [49].

With their high numbers of hydroxyl groups, polysaccharides such as dextran likely behave similarly to PEG in reducing nonspecific interactions of serum proteins with nanoparticles. Dextran is a neutral, biodegradable polysaccharide made up of glucose molecules of 1,6-glycosidic linkages with varying degrees of length and branching. The lower the degree of branching of dextran, the fewer the allergic side effects [50]. Moreover, dextran may have advantages over PEG, since severe allergic side effects may occur less frequently [51,52].

A dextran-b-poly(L-histidine) (Dex-b-PLH) block copolymer was synthesized by Hwang and his colleagues [53]. Poly-L-histidine of two molecular weights (~5800 and ~12,600) was conjugated to the reductive end of the low-molecular-weight dextran (~6000). There were modest differences in the drug loading capacity, pH-dependent size, and release of Dox between these two Dex-b-PLH particles. The release of Dox at both neutral and acidic pH levels from particles containing the higher-molecular-weight (MW) PLH segment was reduced compared to those with the lower-MW PLH segment. This was in contrast to the micelles formed from the diblock polymer (p(HEMA)-b-PLH), in which the higher-MW PLH domain enhanced the release of Dox [47]. Whether this was due to the different block copolymers conjugated to the PLH domain in the two studies is not known. Still, with Dex-b-PLH micelles at pH 7.4, about 40% of Dox was released from the particles with the high-MW PLH in twenty-four hours. Additionally, the cellular uptake rate of Dox-loaded Dex-PLH was higher than that of free Dox, particularly at the lower pH. Consistent with these uptake studies, the cytotoxicity of Dox-loaded particles toward cholangiocarcinoma cells (HuCC-T1) was pH-dependent and greater than that of free Dox [53].

Similarly to dextran, the auricularia auricula polymer (AAP) is a water-soluble polysaccharide that is biodegradable and has potential as a drug carrier. In contrast to dextran, AAP has not been as well characterized as dextran and may be immunogenic [54]. Wang and colleagues formed a nanoparticle named AAP-His by conjugating histidines to a high-molecular-weight AAP. Unlike most polymers described in this section which contain PLH, the hydroxyl groups on the monomeric unit of AAP were modified by a single histidine. The poorly water-soluble drug paclitaxel (PTX) was incorporated within the hydrophobic unprotonated histidine segment of the micelles. The size and the in vitro cytotoxicity of the PTX-loaded AAP-His micelles were pH-responsive. With longer incubation times (seventy-two hours) and at lower drug dosages (0.01 μg/mL), the PTX micelles inhibited the viability of MCF-7 cells to a greater degree than the free drug (MTT assay). The cumulative release rates over a twelve-hour period at pH 7.4 and 5.0 at 37 °C were about 65% and 85%, respectively. If more than a single histidine was conjugated to the monomeric unit of AAP, improved retention at pH 7.4 and greater pH-dependent release of PTX may occur. Notably, in tumor-bearing (sarcoma-180) mouse models, these PTX-loaded micelles significantly inhibited the tumor weight by about 60% more than free PTX (*p* < 0.01) [55]. Although aspects of the design of these AAP-His nanoparticles may be helpful for future drug delivery systems, the use of AAP may be limited because of the induction of cytokines [54]. The CMC was not given in this or the prior dextran study.

It has been suggested that negatively charged nanoparticles have fewer undesirable effects than positively charged nanoparticles. In an interesting report, it was noted that negatively charged micelles at physiological pH that progressively become positive in a slightly acidic environment may target tumors [56]. Specifically, if the charge-reversed micelles become positive (or at least more positive) between 6.5 and 7.0, cellular uptake by tumor cells could be increased significantly. Kim et al. developed one of the two charge-reversed micelles discussed in this review. After the diblock PEG-PAsp (polyaspartic) copolymer was synthesized, 60% of the PAsp groups were modified with imidazole groups (PEG-PAsp-(im). These PEG-PAsp-(im) micelles, formed via the thin-film rehydration method, had zeta potentials ranging from −16 at pH 7.4 to +1 at pH 4.0. Furthermore, the CMC changed dramatically between pH 7.0 and 6.5, going from about 5 to 65 μg/mL. Consistent with the CMC changes, the size increased from 110 to 275 nm between pH 7.4 and 6.5, respectively. As a result, these micelles have the potential to release hydrophobic drugs at pH levels consistent with the extracellular pH levels of tumors. Although no uptake studies were done with these micelles, we think that the less negative zeta potential of the micelles at pH 6.5 would increase their uptake.

One significant problem has been the poor retention of hydrophobic drugs within histidine-containing nanoparticles or micelles at pH 7.4. This was partially addressed in a study by Kim et al. in which a copolymer of histidine and phenylalanine was conjugated to the hydrophilic PEG (PEG-PLH/F) [57]. Notably, the pKa of the polymer varied based on the percentage of phenylalanine and the presence or absence of PEG. As the percentage of phenylalanine increased, the pKa of the diblock PEG-PLH/F decreased. The micelles in which the peptide segment of the block copolymer had a higher molecular weight (M_w_ of peptide: 5600) released about 5% of pyrene after two days at physiological pH. In contrast, the micelles released about 45% and 60% of the pyrene at pH 6.4 and 6.0, respectively. Unfortunately, these investigations do not seem to have explored this particle further. As a result, it is not known whether the release of pyrene from these micelles correlates with the release of Dox, PTX, or other hydrophobic drugs. More studies are needed to discover whether these histidine-containing micelles stably incorporate hydrophobic drugs at physiological pH.

**Figure 3:**
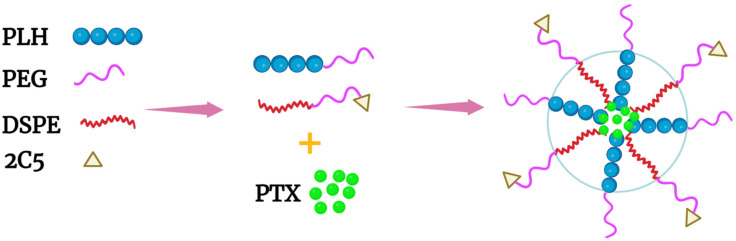
Schematic of a mixed micelle formed with the copolymers PEG-PLH, PEG-DSPE, and 2C5-PEG-DSPE. The hydrophobic drug PTX was incorporated within the inner core, which comprised PLH and DSPE.

Of the co-block polymers, only the PTX-loaded micelles developed by Wu et al. were tested for long-term stability [58]. These mixed micelles, made of mPEG-PLH and PEG-1,2-distearoylphostidylethanolamine (PEG-DSPE) copolymers in a ~1:1 weight ratio, delivered PTX effectively (Figure 2B and Figure 3). Notably, these micelles were stable and released about 10% of PTX at pH 7.4, whereas the micelles released nearly 50% and 65% of PTX at pH 6.0 and 5.0, respectively, over the same time period (twenty-four hours). The release of PTX at pH 5.0 was dramatic, with a burst release of 60% (twelve hours). Consistent with the PTX release data, 4T1 cells incubated in media at pH 5.8 were very sensitive to the cytotoxic effects of the PTX-loaded micelles compared to cells incubated in media at the same pH with free PTX. Since the pH 5.8 medium had no cytotoxic effect on 4T1 cells, the toxicity was attributed to the PTX or the PTX-loaded mixed micelles. Moreover, the inclusion of an antinucleosomal antibody (2C5-PEG-DSPE) on the surface of the micelles further enhanced their cytotoxicity. Notably, with PLH and DSPE forming the hydrophobic core, the PTX-containing micelles were stable for several months at 4 °C. Since these micelles have several attractive properties, this formulation deserves further study. However, because of the size of the 2C5 monoclonal antibody and its possible lack of tumor specificity [59], single-chain antibodies, as well as other small tumor-specific ligands, should be investigated with this mixed micelle preparation.

**Figure 4:**
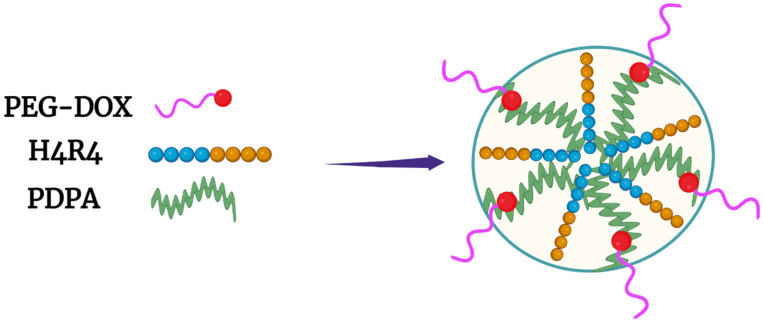
Micelle formed with PEG-Dox conjugate and the hydrophobic PDPA polymer. The H4R4 peptide enhanced endosomal lysis, increasing Dox release into the cytosol.

Another promising approach for delivering Dox was reported by Liang et al., who entrapped a histidine–arginine co-peptide within their NPs [8]. The PEG-Dox conjugate, together with the hydrophobic PDPA polymer (also named PDPAEMA), was mixed with various ratios of the H4R4 co-peptide (HHHHRRRR) (Figure 4). The H4R4 was incorporated into the NPs to enhance endosomal escape/lysis of the PEG-Dox conjugate. Importantly, the H4R4 did not affect the release of the PEG-Dox conjugate. The PDPA, with a pKa of 6.4, was the primary factor in disrupting the NPs and releasing the H4R4 co-peptide and the PEG-Dox conjugate. At a weight percentage of 14%, the incorporated H4R4 increased the cytotoxicity 30-fold compared to the Dox-loaded NPs. This underscores how vital endosomal lysis was in enhancing the efficacy of Dox. Notably, the Dox-loaded NPs were quite stable and released about 10% of the Peg-Dox at pH 7.4, while the NPs released about 90% of the PEG-Dox at pH 5.5 over the same time (thirty-six hours). It is likely that most of the Dox was bioavailable from the PEG-Dox conjugate, since the Dox-loaded NPs showed markedly more cytotoxicity than the free Dox toward HeLa cells (IC_50_: 0.063 vs. 1 μM). An interesting comparison would perhaps be to examine the non-pH-dependent amide bond between PEG and Dox in this study using a pH-dependent linkage [60]. Nonetheless, the amide bond seemed to be readily cleaved in HeLa cells.

Several questions arise from this study. Would incorporating the H4R4 peptide into histidine-rich or other pH-dependent micelles enhance cytosolic delivery of the drug? Since the R4 peptide was likely on the surface, would a longer histidine segment add greater stability to the micelle? Why did the pH-sensitive PDPA not effectively lyse endosomes? It is of note that most block or grafted PLH polymers discussed in this review were not tested for their endosomal lysis potential.

## 3. Branched Polymers

The spectrum of nanoparticles formulated using branched polymers was similar to that of those made using linear block polymers. Most of the nanoparticles comprised a diverse group of branched copolymers (i.e., graft copolymers or star-shaped polymers) with a pH-dependent disrupting histidine component. In some cases, the nanoparticles formed of graft polymers were stabilized by coating the surface with a pH-dependent polymer. Although it was not clear whether the stability of the drug-loaded nanoparticles was greater with the use of the branched polymers, a polymeric graft micelle showed marked stability when PLH was the sole component of the hydrophobic domain [61].

Tsai et al. examined multifunctional micelles comprising a graft p(HEMA-co-histidine)-g-PLA polymer and a diblock polymer, folate (FA)-PEG-PLA [62]. The graft copolymer was designed to be pH-sensitive and to encapsulate Dox, whereas the FA-PEG-PLA formed a hydrophilic shield and the targeting component. In HeLa cells, the folate–micelles loaded with Dox had higher uptake and inhibited the cells significantly more than the untargeted micelles. Moreover, the researchers observed that a pH change led to a significant release of Dox, owing to the buffering capacity of the histidine components. Notably, after the initial burst of 10% release of Dox from the micelles in the first two hours, minimal release of Dox from these micelles occurred over the next three days at pH 7.4 and 37 °C. In contrast, about 50% of Dox was released from the micelles in the first twelve hours at pH 5.0, with a cumulative release of 65% over three days. Furthermore, infrared imaging of HeLa-bearing mice demonstrated that the targeted micelles accumulated in tumor xenografts two-fold more than untargeted micelles. Consistent with these findings, the targeted Dox micelles significantly inhibited tumor size in a mouse model compared to the untargeted micelles or Dox-alone therapies. These findings indicate that multifunctional theranostic micelles could detect tumors via imaging and deliver sufficient antitumor drug doses without systematic toxicity [62]. A similar but more recent study showed that these Dox-loaded micelles effectively inhibited Lewis lung cancer xenografts in a mouse model [63].

**Figure 5:**
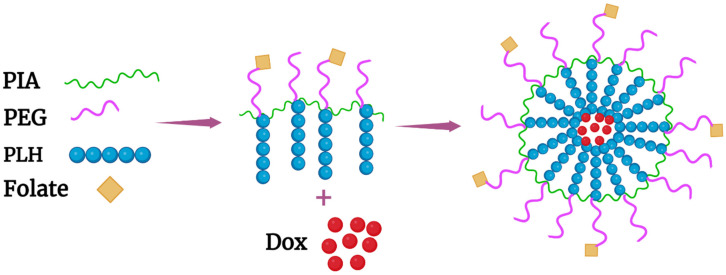
Schematic of the targeted graft polymer PIA-g-PEG-FA-g-PLH and the formed micelle. The pH-sensitive hydrophobic PLH domain entrapped Dox, whereas the folate-PEG ligand was on the exterior of the nanoparticle.

**Figure 6:**
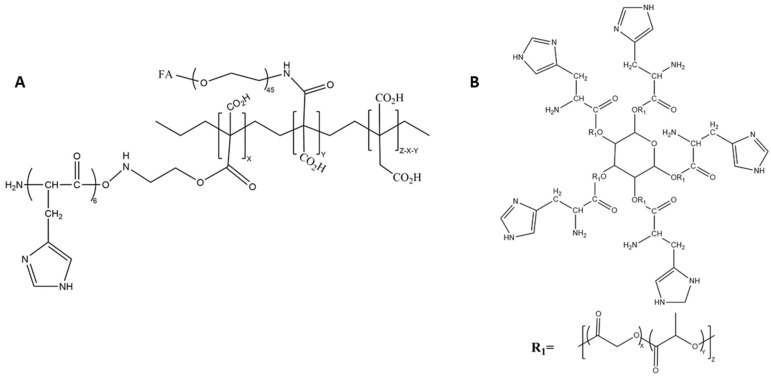
Chemical structure of the (**A**) graft polymer PIA–g-PEG–FA–g-PLH, and (**B**) the five-armed star-shaped branched PLGA copolymers in which a single histidine was coupled to the end of each arm.

Probably the most stable and pH-responsive micelle described in this review was that comprising a graft PEGylated copolymer with a folate ligand (poly(itaconic acid)–g-poly(ethylene glycol)–folate–g-poly(L-histidine) (PIA–g-PEG–FA–g-PLH) [61]. Polyitaconic acid (PIA) was the backbone onto which the PEG-FA and PLH polymers were conjugated (Figure 5 and Figure 6A). This was the sole drug-laden nanoparticle (micelle) described in this review that was extremely stable at pH 7.4, and in which only PLH formed the hydrophobic core. Specifically, about 2% of the Dox was released by forty-eight hours at pH 7.4 and 37 °C, whereas 65% and about 90% were released by twelve and twenty-four hours, respectively, at pH 5. Moreover, there was a graded release of Dox (~20% by twenty-four hours) from the micelles beginning at pH 7.0. Notably, there was a charge reversal of these micelles upon lowering the pH. The zeta potentials of these micelles were −16, −5, and +14 at pH 7.40, 6.50, and 5.0, respectively. In the cellular uptake experiments, the FA-targeted micelles had higher fluorescence intensity than nontargeted micelles. Moreover, Dox-loaded PIA-g-PEG-FA-g-PLH micelles showed greater inhibition of HeLa cells than free Dox in a pH-dependent manner. Unfortunately, in vivo studies were not done with these promising Dox-loaded micelles [61].

In contrast to many other studies described in this review, one group developed a micelle preparation in which the histidine content of the polymer had no primary role in forming the hydrophobic center [64]. Yang et al. directly conjugated histidine and the hydrophobic C18 acyl chain to poly(2-hydroxyethyl aspartamide) (PHEA-g-C_18_-His) to form micelles. However, as the number of histidines conjugated to the primary PHEA chain increased, the size and zeta potential of micelles increased as the pH was lowered. The release of Dox from the PHEA-g-C18-His micelles was modestly pH-dependent, with a 15% difference in release between pH 7.4 and 5.0 (over twenty-four hours). It should also be noted, however, that nearly 50% of the Dox was not released from the micelles after three days at pH 5.0, suggesting that these micelles were too stable. Nevertheless, significant amounts of Dox from the His-containing micelles were observed in the nucleus within twelve hours, whereas Dox from the non-His micelles was limited to the endosomes. Consistent with these findings, the PHEA-g-C18-His micelles loaded with Dox inhibited the tumor cells markedly more than the non-histidine-containing micelles. Although the Dox-loaded micelles showed greater cytotoxicity at early time points, free Dox over a wide range of concentrations was more effective in reducing the cellular viability at twenty-four hours.

**Figure 7:**
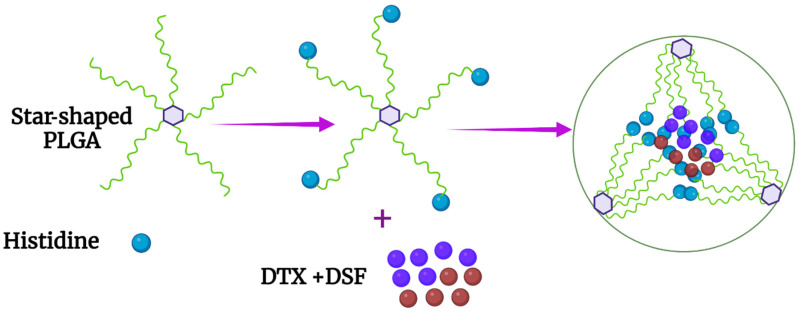
Schematic of the five-armed star-shaped polymer in which PLGA-His forms the inner shell. The hydrophobic drugs docetaxel and disulfiram, which have demonstrated antitumor synergy, were entrapped within the interior of the micelle.

To develop a histidine-modified nanoparticle (NP) that would release hydrophobic drugs at pH 6.5, consistent with the T_E_, Swetha et al. synthesized a five-armed star-shaped PLGA polymer in which a single histidine was coupled to each arm’s end (Figure 6B and Figure 7) [65]. Based on a previous study showing antitumor synergism between DSF (disulfiram) and DTX (docetaxel) [66], nanoparticles loaded with these drugs were formed via the application of microfluidic technology using the nanoprecipitation method. The researchers had previously determined that DSF and DTX were synergistic in killing cancer cells. While the size of nonhistidine PGLA nanoparticles (NPs) was not affected by pH, the histidine (His)-containing particles increased markedly in size from 157 nm at pH 7.4 to 1268 nm at pH 6.5. Consistent with the increase in size, the release of both drugs was pH-dependent. While about 20% of the drug was released from the His-modified NPs at pH 7.4, about 60% was released at pH 6.5 over the same twelve-hour time period. The star-shaped micelles, which incorporated DTX and DSF, showed significantly greater cytotoxicity toward MCF-7 spheroids than the free drugs. Importantly, using tumor spheroids as a surrogate for tumors, the pH-sensitive particles had greater penetration than non-His particles. Specifically, in spheroids, which have an acidic extracellular pH, the His-PGLA nanoparticles showed penetration of 200 μm into these nonvascular models. Consequently, these nanoparticles should invade far into the hypoxic areas of tumors, which begin approximately 70 μm from normal vessels [67]. Based on prior studies, the mechanism of the greater penetration into the spheroids may have been due to the interaction between the protonated pH-sensitive polymers of the micelles and the cell membranes in the superficial layer, resulting in cell death and enabling greater exposure of cells in deeper layers to micelles [68,69]. Deeper penetration into tumors via this mechanism likely applies to several histidine-containing NPs described in this review and this greater penetration may circumvent one of the major therapeutic obstacles of nanoparticles (see “Challenges and Future Directions” section for further discussion).

The polysaccharide hyaluronic acid (HA) has been modified with histidines [70] or polyhistidine to form micelles [71]. In the latter paper, the hydrophobic PLH was grafted to the negatively charged hydrophilic linear HA polysaccharide to form copolymers (HA-g-PLH) [71]. HA targets the overexpressed surface receptor CD-44 on cancer cells, and the negative charge of HA likely improves the half-life and biocompatibility of nanoparticles. Consequently, Qiu et al. varied the degree of substitution (DS) on the HA molecule from 19 to 28 (HA-g-PLH-19, 22, 28) using PLH peptides. With greater degrees of substitution, the particles decreased in size, probably due to enhanced hydrophobic packing and a reduction in the number of carboxyl groups on the HA polymer. Nevertheless, the DS had to be balanced with a reduction in the carboxyl groups on HA, which were necessary for the uptake of the HA-g-PLH micelles via CD-44-mediated endocytosis. The authors determined that the Dox-loaded micelles with the lowest DS (HA-g-PLH-19) were the most effective in enhancing the uptake and reducing the viability of MCF-7 cells. Interestingly, the IC_50_ of the Dox-loaded HA-g-PLH-19 was 1.76 for MCF-7 cells, which was lower than that of free Dox (IC_50_: 2.4). As expected, the release of Dox was pH-dependent, but the release of Dox at pH 7.4 was significant (35%) over a twelve-hour period. Compared to several other micelles, the CMCs of these hyaluronic-acid-based micelles modified with histidines or PLH ranged from 20.4 to 39 μg/mL [70,71], suggesting that their stability in the bloodstream may be an issue.

Most nanoparticles, including the HA particles discussed in the preceding paragraph, expose the targeting ligand on the particle’s outer surface. Although this strategy may increase accumulation in tumors, these particles may also target normal tissues that express the receptor. To avoid this potential problem, Huang et al. developed HA-decorated nanovesicles that were cloaked until the particles were exposed to lower pH levels [72]. Once the HA was exposed, uptake by CD-44-expressing tumor cells and tumor-associated macrophages (TAMs) occurred. In addition to killing tumor cells, the drug-loaded nanovesicles used TAMs to hitchhike their payloads to the hypoxic areas of the tumor. The nanovesicles were formed from a graft copolymer, HA-g-PLGA, and the vesicles encapsulated the chemotherapy drug 7-ethyl-10-hydroxylcamptothecin (SN38). These vesicles were further sealed with a pH-sensitive methoxy-pegylated-b-p(histamine-methylacrylamide) (mPEG-pHPMA) polymer. Upon exposure to a pH of 6.7, compatible with the extracellular tumor pH, the nanovesicles showed reduced stability and smaller size, consistent with removal of the pH-sensitive polymers from the nanovesicles. With the HA then exposed at lower pH levels, these nanovesicles showed enhanced uptake and cytotoxicity in the CD-44-expressing prostate cancer and TAM cells. Concomitant with the deeper penetration of the mPEG-HPMA-coated nanovesicles within Tramp1 tumors, the vesicles loaded with SN38 showed significantly greater tumor inhibition than the noncoated vesicles or the pH-insensitive coated vesicles. Because the imidazole (histamine)-containing polymer was removed before endocytosis, the pH-dependent release of SN38 was not investigated. We revisit this method of shrouding the ligand in the “Composites” section of this review.

## 4. Dual Delivery of Chemotherapy and Nucleic Acid

Both linear block and graft polymers have been used to design nanoparticles for the dual delivery of chemotherapeutic agents and siRNA. While the hydrophobic agents are almost always in the interior, nucleic acids may be located in the interior or exterior, depending on the design of the particle. This is true for histidine-containing or other pH-sensitive nanoparticles [73,74,75]. Although siRNA alone is hydrophilic, its negative charge can be neutralized, increasing its hydrophobicity. The in vivo antitumor activities of several of these dual-delivery nanoparticles are quite remarkable.

**Figure 8:**
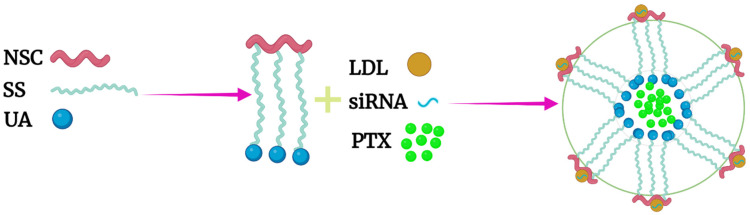
Dual pH- and redox-responsive micelles were formed from the binary polymer of LDL and N-succinyl chitosan-cystamine-uronic acid (LDL-NSC-SS-UA). The LDL ligand on the exterior of the micelle binds tightly to the cholesterol-modified siRNA. In contrast, PTX was located in the interior and was released by redox and pH stimuli.

For the dual delivery of histidine-containing NPs, the outer shell of the NPs developed by Zhu et al. consisted of the binary polymer of chitosan and the targeting lipoprotein LDL, which binds to cholesterol-modified siRNA. The multilayered siRNA-PTX-loaded micelle demonstrated antitumor activity against drug-resistant MCF-7 cells in vitro and in vivo (Figure 8) [49]. The siRNA targeted the breast cancer resistance gene BCRP, which is important for the efflux of chemotherapy drugs such as PTX. The micelles were formed by film dispersion of the binary polymer in which the primary hydrophilic chain was chitosan, and the hydrophobic branches had both a disulfide bond and a terminal imidazole end group (uronic acid). As a result, the release of PTX from the inner hydrophobic core of the micelles was redox- and pH-dependent. Despite the relatively modest pH- and redox-responsive release of Dox from these micelles, the targeted therapy showed marked antitumor synergism of the siRNA and PTX therapeutic agents in vivo against the drug-resistant cancer xenografts. Indeed, there was little to no growth of the tumor xenografts over the 20-day treatment period.

A pH- and redox-sensitive nanoparticle was also developed to deliver Dox and a siRNA targeting the MDR1 gene [76]. The nanoparticles were composed of PEG-b-PLA-PLH-ss-OEI (oligoethylenimine) polymer, and the particles were made via the thin-film rehydration method. While PEG formed the hydrophilic shell, the PLA and PLH domains formed the hydrophobic interior that incorporated Dox. In contrast to other nanoparticles in which a nucleic acid was included on the outer surface [74,77], the siRNA, being neutralized by the positively charged OEI polymer, was in the inner cavity of the micelle. A specific N/P ratio (7:1) between the OEI and siRNA was established and was critical for efficient silencing. As far as stability, the nanoparticles released about 35% of Dox within twelve hours at pH 7.4, while they released 70% and 89% of Dox at pH 6.5 and 5.5, respectively. Although the release of Dox was not redox-dependent, the release of the siRNA was both pH- and redox-dependent [76]. In vivo studies were particularly impressive, showing that these nanoparticles virtually inhibited the growth of drug-resistant MCF-7 xenografts.

To mitigate the toxicity caused by fully protonated polyamino acids such poly-L-lysine, Wahane et al. compared different formulations of PLGA and histidines to incorporate the antitumor agents paclitaxel or a peptide nucleic acid which targeted miR-155 (PNA-miR-155) [78]. Along with the hydrophobic drugs, their double-emulsion evaporation formulations included PLGA, histidine-modified PLGA, and PLH and PLGA. In contrast to other formulations, the NPs of PLH mixed with PLGA had a positive surface charge of about +20 in water (these may be less positive in physiological media). Consequently, PLH/PLGA amalgam NPs with their positive charge had a higher cellular uptake than the negative-surface-charge NP formulations. Notably, this was the only nanoparticle we could find in which PLH was incorporated on the outer surface of a polymer-formed particle. Although the pH dependency of drug release was not investigated, the PLH/PLGA NPs carrying either PNA-miR-155 or PTX markedly inhibited lymphoma cells in vitro and in vivo. Notably, PTX and miR-155 were incorporated and their efficacy evaluated separately, suggesting the possibility of combining them for enhanced therapeutic activity. Because the release of the neutral PNA from the PLH/PLGA NPs was about 50% over one hour and nearly 100% over twelve hours at pH 7.4, greater stability of these NPs will be required.

Another approach has been to incorporate the negatively charged methotrexate (MTX) within cationic lipopeptides to form PEGylated nanoparticles targeting the EGFR receptor [79]. Unlike the other polymers described in this review, this branched lipopolymer and the subsequent peptide carriers described in this section were made using a peptide synthesizer. These branched cationic lipopeptides containing histidines were effective dual carriers of MTX and a siRNA targeting the tumor survival gene EG5. Moreover, further modifying MTX with a higher number of carboxyl groups increased the stability of the nanoparticles in serum. The EGFR-targeting lipopolymer incorporating siRNA and the modified MTX inhibited metabolic activity effectively and increased apoptosis in MTX-resistant KB cells. All three components, the EGFR-targeting peptide, the siRNA, and the modified MTX, played key roles in reducing the viability of the cells. Together with the ethylenediamine groups, the incorporated histidines likely had several functions, including disruption of the nanoparticles and increase of endosomal lysis.

With low efficacy reported particularly in human clinical trials, new approaches are required to enhance the uptake of nanoparticles by tumors [80,81]. The neuropilin-1 receptor is frequently upregulated in tumor endothelial and tumor cells; this receptor recognizes the cognate ligand -KXXK, where “X” is any amino acid. To take advantage of this active tumor transport system, we synthesized a linear histidine-rich peptide, H2K, with a primary sequence of -KHHK-. Because DNA binds with high affinity to Dox [82,83], an H2K-DNA-Dox polyplex was formed that markedly reduced the growth of tumor xenografts compared to free Dox [84]. Although the DNA in this study did not express a tumor suppressor protein, preliminary data showed that H2K-DNA-Dox could express luciferase (Luc), and antitumor synergism between p53 and Dox may occur. However, because Dox intercalates with plasmid DNA and can limit the expression of proteins, the amount of Dox loaded into the polyplex may be limited.

Similarly, Li et al. developed nanoparticles that targeted the NRP-1 receptor in tumor xenografts. They co-delivered a siRNA targeting PD-1 and a small-molecule checkpoint inhibitor, methyl-dl-tryptophan, in micelles to reduce tumor size [81]. The micelles were composed of an NRP-1 tumor-targeting peptide with a histidine-rich domain and cholesterol. The micelle showed modestly pH-sensitive release of methyl-dl-tryptophan. Nearly 50% of the molecule escaped from the micelles at pH 7.4 within forty-eight hours, whereas about 75% escaped at pH 5.0. Compared to either checkpoint inhibitor alone, co-delivery of the siRNA and 1-methyl-dl-tryptophan induced marked reduction in the size of 4T1 tumors.

## 5. Composites

In contrast to the previous sections, this section examines the interactions between imidazole-containing polymers and nonpolymer nanostructures. The imidazole polymers may form particles that incorporate smaller nanostructures within them, or coat the surface of nanoparticles such as mesoporous silica nanoparticles (MSNs). Although MSNs offer several advantages, such as a stable structure and excellent biodegradability, MSNs tend to aggregate when exposed to biological media, resulting in the release of drugs. Coating these MSNs with polymers minimizes these problems and blocks the release of drugs entrapped within the pores of the MSNs. To release drugs from MSNs precisely, pH-responsive polymers such as poly(2-diethylaminoethyl methacrylate (PDEAEMA) [85,86] and polyhistidine [87,88] polymers have been used to coat MSNs.

Bilalis et al. modified and compared MSNs decorated with two different molecular weights (6.8 and 4.5 kDa) of PLH [87]. It is of note that the protective trityl group on PLH could be gently removed by treatment with trichloroacetic acid. The PLH-coated MSNs showed an increase in size and zeta potential as the pH was reduced to 5. Importantly, these polymer-coated MSNs were stable in serum for twenty-four hours. Although both PLH-modified MSN preparations showed similar pH-dependent release of Dox, the release of Dox from the higher-molecular-weight polyhistidine MSNs at physiological pH was significantly lower. Approximately 10% of the drug was released at pH 7.4 from the higher-molecular-weight PLH-MSNs, while about 50% was released at pH 5.0 over seventy-two hours. Most of the pH-related release of Dox occurred within the first five hours, and this raised concerns about whether the 50% of Dox remaining would be released.

In a recent study, a PEGylated and PLH-modified MSN particle released sorafenib in a pH-dependent manner, and these PEG-PLH-MSNs markedly inhibited the size of hepatomas when systemically delivered. The PEG-PLH-MSNs modestly inhibited the tumors more than MSNs without PLH (PEG-MSN) [88].

Although liposomes are the only carrier of Dox approved by the FDA to treat human cancer, there is only one histidine-coated polymer (PHMA) liposome preparation that incorporates Dox. Because there have been reports that Doxil^®^ may not sufficiently release Dox [89], these histidine-containing polymeric liposomes offer the opportunity to facilitate this drug’s release. One potential problem is that the Dox released inside the more acidic endosomes will become more protonated and thus less able to cross the endosomal membrane to reach the cytosol [90].

To tackle this problem, Chiang et al. developed a polymer-coated liposome that targeted the acidic extracellular tumor environment [91]. They synthesized a PEG-b-(HPMA-co-histidine)-cholesterol polymer in which the hydrophobic cholesterol enabled the insertion of the polymer into the liposomal membrane. These liposomes also contained a cloaked biotin ligand (biotin-PEG-biotin) targeting its receptor, which is commonly upregulated in many cancer cells, to enhance uptake into the cancer cells [92]. To reduce drug leakage, these liposomes were sealed through the interaction between the biotin-PEG-biotin polymer and the blocked HPMA-co-histidine copolymer. At pH 7.4, Dox leakage from these liposomes (ECM-liposomes) was about 20% after a twenty-four hour incubation with albumin. In comparison, about 40% of Dox was released from the nonsealed liposomes. At pH 6.8, at which point the histidine became partially protonated, the cloaked biotin was exposed and uptake of the ECM-liposomes into the cancer cells was greatly enhanced. Although the pH-dependent release of Dox was not examined using liposomes not containing the biotin polymer, increased leakage might occur at a lower pH because of liposomal disruption by the charged histidine-containing polymer. Notably, these ECM-liposomes, delivered intravenously, displayed significantly greater accumulation in HCT116 tumor xenografts than the non-biotin-containing liposomes. Despite these encouraging results, the cytotoxicity and in vivo inhibition of these ECM-liposomes toward colon cancer cells or xenografts were not examined.

Most of the micelles discussed in this review have been developed for systemic delivery to target tumors. These therapies avoid several obstacles, including the lung capillaries, excretion by the kidneys, opsonization with proteins such as albumin, and uptake by large organs such as the liver. Local delivery of these therapies avoids many of these obstacles and increases the amount of therapy that reaches the tumor. One such local therapy is transcatheter embolization to deliver chemotherapeutics to hepatic cancers that cannot be resected [93]. To embolize an orthotopic hepatic carcinoma in a mouse model, microspheres with an average size of 5 μm were delivered into the hepatic artery using an intra-arterial catheter. The PLGA microspheres contained an imidazole-functionalized polypeptide with a lipid tail (C18-PLGA-PAA_10_-g-Im) and the hydrophobic multikinase inhibitor sorafenib, as well superparamagnetic iron oxide nanocubes (25 nm in size) for imaging. When delivered via the catheter to the acidic environment of the tumor, the embolic pH-sensitive microspheres significantly enhanced apoptosis compared to the pH-insensitive microspheres. Moreover, the dual-functioning nanoparticles could be visualized within the tumor using magnetic resonance imaging.

## 6. Challenges and Future Directions

A significant obstacle associated with systemic NP delivery is that less than one percent of a nondirected nanoparticle preparation accumulates in the tumor [94]. Therefore, several approaches with nanoparticles are being explored to specifically direct and release the therapy in tumors to achieve higher accumulation in tumors. A promising approach has been stimulus-based therapies, including the release of chemotherapeutic agents from nanoparticles in the acidic environments of the tumor matrix or the tumor endosomes.

Nanoparticles and the drugs they carry are exposed to dramatically different pH ranges once they leave the blood vessels that feed the tumor. Two of these, the T_E_ and the more acidic endosomes of tumor cells, are acidic, whereas the cytosol of tumor cells is relatively alkaline (pH 7.15 to 7.44) compared to the T_E_. [28,95]. Consequently, pH-dependent nanoparticles are being developed for their ability to release drugs intracellularly or extracellularly in tumors.

Those micelles developed to release low-molecular-weight drugs in the T_E_ have greater potential to penetrate tumors and reach a larger number of cells. Several modifications of polymeric nanoparticles have been used to release drugs between pH 6.5 and 7.0, the range of pH in the T_E_. These include blending PLH with PLA domains, adding PEG to PLH, polymerizing a mixture of histidines and phenylalanine, and altering the copolymer structure (i.e., star-shaped copolymers) [10,39,57,65]. Still, micelles that release a large percentage of the loaded drug extracellularly depend on intracellular uptake for the complete release of the drug. With future advances, greater selectivity in the release of drugs between these two acidic compartments is anticipated. Although a couple of nanoparticles comprised of copolymers became smaller in size [10,72], most NPs became larger as the pH transitioned from 7.4 to 6.5. This increase in their size may reduce the particles’ ability to invade deeper into solid tumors.

Nevertheless, some pH-sensitive nanoparticles showed deeper invasion into tumor spheroids, a surrogate for tumor penetration, than non-pH-sensitive NPs [65]. The greater penetration into spheroids may have been due to the interaction between the charged pH-sensitive polymers of the NPs and the cell membranes in the superficial layer, resulting in cell death and enabling greater exposure of cells in deeper layers to the NPs. Alternatively, to circumvent the inability of nanoparticles to penetrate into tumors, the micelles developed by Huang et al. combined endosomal uptake by TAMs with the subsequent release of SN38 into the deeper parts of the tumor [72]. Upon exposure to mildly acidic pH environments, the histidine-containing polymer cloaking the nanoparticle was released, exposing the HA ligand on the nanoparticle. Subsequently, the uptake of drug-loaded micelles by TAMs resulted in the transport and release of SN38 into deeper parts of the tumor. Surprisingly, the release of SN38 by TAMs showed cytotoxic activity against the tumor cells. With the addition of the HA ligand to improve uptake by TAMs and cancer cells, this approach could be applied to other micelles. Other potential targets on TAMs and tumor cells include legumain and the p32 receptor [96]. Notably, both lytic and hitchhiking histidine-containing nanoparticles can penetrate 200 μm into spheroids or tumors, significantly beyond the 70 μm distance from normal vessels at which hypoxia begins [67].

Although most studies showed a pH-dependent release of the drug, significant drug retention in the nanoparticles is also a concern. For example, nearly 50% of Dox was retained after three days in one micelle [64]. Without the complete release of Dox, the antitumor efficacy of the nanoparticle is, of course, limited. Conversely, if a significant amount of drug is released at pH 7.4, then the pH specificity of the drug-laden nanoparticle will be decreased, and greater toxicity to normal tissues may be observed. A high degree of release of hydrophobic drugs at physiological pH was frequently observed with the block copolymer nanoparticles [45,46,55]. To stabilize NP–drug interactions, adding a pH-sensitive bond, enhancing the hydrophobicity of the PLH domain (e.g., by using a mixture of phenylalanine/histidine), or increasing the size and hydrophobicity of the drug may reduce leakage of the drug. These stratagems have been inadequately explored for histidine- and PLH-containing nanoparticles [57,97,98]. Notably, several micelles/nanoparticles were relatively stable at pH 7.4 and released significant amounts of the drug at lower pH levels [8,58,61,62]. However, even these stable nanoparticles, particularly the micelles, may not be stable when translated to human trials with a significantly larger blood volume, and cross-linking agents may be required [99].

A related issue is the shelf-like stability, which may be a problem with some particles. Although retention experiments for chemotherapy agents are usually done at 37 °C and 100 RPM, Oh et al. indicated that micelles did not have long-term stability, presumably at a lower temperature [42]. Micelles/nanoparticles stored at 4 °C would be more stable than those stored at higher temperatures and could be used within twenty-four hours of reconstitution, assuming lyophilization can be achieved successfully. Of the multiple studies we reviewed, a single study commented on whether their micelles had long-term stability [58]. In addition, only one paper detailed whether the lyophilization process affected the size, charge, and biophysical properties of the nanoparticles/micelles [42]. In this case, the investigators reconstituted the drug-loaded micelles while maintaining their biophysical and antitumor efficacy by adding 33 wt% Pluronic F127 to the micelles. Clearly, more studies are required to determine whether lyophilization can be done without modifying micelles.

Strategies to take full advantage of modifying tumor pH depend on the chemotherapeutic agent and whether the agent will be released extracellularly or within the endosomes. For example, raising the extracellular pH of tumors with bicarbonate enhanced the uptake and efficacy of Dox in tumor cells [90]. Although administration of bicarbonate to disrupt NPs in the T_E_ is not desirable, modestly raising the endosomal pH of tumor cells with histidine-rich buffering carriers may similarly increase the transport of Dox into the cytosol. Alternatively, if an endosomal lysis peptide such as R4H4 is included within the NPs [8], Dox may more readily escape into the cytosol. In contrast to raising the pH of the endosomes, lowering the extracellular pH of solid tumors via the administration of glucose may, in general, be advantageous for disrupting a pH-dependent nanoparticle preparation [22,100], but particularly for a nanoparticle preparation delivering a derivative of camptothecin extracellularly. A lower pH can prevent the lactone ring of camptothecin or its analog from opening, thus reducing its inactivation [38]. Notably, one study indicated that tumors did not become more aggressive upon administration of glucose [101]. Still, more studies are required to determine whether intermittent reduction of tumor pH will enhance malignant and metastatic potential.

There are many promising yet incompletely investigated nanoparticles discussed in this review. Some of these nanoparticles have only had their biophysical properties characterized without in vitro or in vivo studies done [57,87]. Other nanoparticles have shown promise regarding their stability, pH responsiveness, and in vitro cytotoxicity, but in vivo studies have not been initiated [8,44,58,61]. Still others have shown marked antitumor activity, but their stability at physiological pH may be problematic, and further stabilization of these NPs may be required [46,76]. Of the 37 nanoparticles covered in this review, six studies examined the in vivo biodistribution of drug-loaded nanoparticles [40,41,46,62,72,76]. In addition, only one study examined the pharmacokinetics of their nanoparticle [40]. While potential methods exist to stabilize nanoparticle–drug interactions, modifications of NPs to increase the half-life cannot be done until baseline studies are completed.

## 7. Conclusions

This review highlights the diverse number and designs of pH-sensitive histidine-containing carriers for enhanced delivery of cancer therapeutics. The histidine-containing polymers include linear polymers conjugated to histidines or imidazoles, linear histidine-containing peptides, diblock and triblock peptides/polymers, graft polymers, and star-shaped copolymers. These polymers formed micelles, mixed micelles, nonmicelle nanoparticles, nanovesicles, lipopolyplexes, and polyplexes, with micelles being predominant. The described diversity also includes tumor-targeting ligands attached to these nanostructures, most of which improved antitumor efficacy significantly. Of the hydrophobic drugs Dox, PTX, 7-ethyl-10-hydroxylcamptothecin, and 1-methyl-dl-tryptophan, Dox was the most common drug, incorporated into 66% of particles. While Dox levels extracted from tumors can be measured fluorescently [84], the second most incorporated drug, PTX, may have its intratumoral levels measured using HPLC [102]. More recently, the distribution of PTX within tumors and tissues has been determined using MALDI mass spectrometry imaging [103]. While Pi-Pi and hydrophobic bonding between the carriers/NPs and these drugs were the prevalent interactions, ionic bonds still played an essential role in the Dox and MTX polyplexes [79,84].

Consistent with changes in the zeta potential and size of the histidine-rich nanoparticles, most of the drugs were released in a pH-dependent manner. In several cases, the drug-loaded nanoparticles/micelles were significantly more effective than the free drug in reducing the size of tumors in animal models. At least in the short term, the drug-loaded NPs showed low toxicity compared to the conventional free drugs. Consequently, the therapeutic windows for these drug-loaded micelles may be much greater than those for the free drugs. Although the stability and retention of the chemotherapy agent was an issue with many NPs, several promising drug-loaded nanoparticles were stable and released most of the drug at a lower pH [8,58,61,62].

In addition to delivering single agents, dual delivery of nucleic acid inhibitors and chemotherapy by nanoparticles offers the potential to synergistically inhibit tumors. An unmet need in chemotherapy is for strategies to address the frequent observance of drug resistance. Pump efflux mechanisms such as the MDR1 and BCRP transporters decreased cells’ levels of these chemotherapy agents. With micelles, the dual delivery of Dox or PTX and siRNA inhibitors targeting the efflux transporters showed marked synergism in the antitumor activity in tumor-bearing animal models. Alternatively, lipopolyplexes showed synergistic antitumor activity upon delivery of two chemically distinct inhibitors. One of these lipopolyplexes contained methotrexate and a siRNA inhibiting a cell survival protein, whereas the other delivered a siRNA and methyltryptophan, targeting different checkpoint inhibitors, and demonstrated marked antitumor activity in vivo.

As stated previously, one report indicated that less than 1% of the nanoparticle dose accumulated within the tumor. However, other investigators have suggested that the accumulated dose may be much higher and that more traditional methods (e.g., AUC) may be more predictive of accumulation and therapy [104]. Maeda recently suggested that criticism of the EPR effect was unfounded, and that the low accumulation and lack of antitumor efficacy were due to the short half-lives of most of the developed nanoparticles [105]. Somewhat supporting this is the fact that a well-characterized PTX-micelle with a long half-life had an accumulated dose that correlated with the antitumor activity [106]. Interestingly, after multiple doses, the % ID (percent injected dose) was significantly enhanced with the higher PTX micelle dosage, indicating that the EPR effect is dynamic and responsive. Adding to the complexity, however, a recent report based on gold particles suggested that an active transport system and not EPR was the primary entry for nanoparticles into tumors [107]

Regardless of the mechanism of entry, a half-life sufficient for a nanoparticle to reach its target would seem necessary for its efficacy. Nevertheless, defining a sufficient half-life may depend on the type of particle, the mechanism of entry, and the blood volumes and circulatory times of the animal. As we have discussed, few pH-dependent histidine particles with promising tumor inhibition have been subjected to pharmacokinetic or biodistribution studies. Since the antitumor effectiveness of therapies can frequently vary dramatically between mice and humans, it is essential to compare PK and target studies done in small animal models and humans. Built on such published results, iterative redesigning of the polymers and nanoparticles will hopefully facilitate the development of a field that has somewhat languished. Alternatively, if the nanoparticles do not accumulate efficiently in tumors in humans, then other approaches should be tried, such as targeting the active NPR-1 transport system.

**Table 1:** Selected pH-sensitive and drug-loaded NPs.

Polymer	Drug	Cell Lines (In Vitro, In Vivo)	Comments	Reference
**Linear Block and Imidazole Polymers**				
PEG-b-PLH(75%)/ FA-PEG-b-PLL (25%) ^1^	Doxorubicin	MCF7/MCF7^R^ (+,+) ^2^	Mixed micelles formed. Folate ligand markedly improved antitumor activity in vivo of Dox-loaded micelles against MCF7^R^ cells. Mixed Micelles released 45% more Dox ^3^ at pH 6.8 than at pH 7.4 (24 h). Biodistribution studies were done.	Lee et al., 2003 [39], 2005 [41]
PLA-PEG-PLH	Doxorubicin	MCF7 (+,−)	Flower-like micelles formed. Micelles released about 30% more Dox at pH 6.8 than pH 7.4 (24 h). Dox-loaded micelles showed progressively greater toxicity toward cells in vitro as pH was lowered.	Lee et al., 2007 [43]
mPEG-PLA-PLH	Doxorubicin Resveratrol	MCF7^R^ (+,+)	Resveratrol inhibited MDR transporter. Dox release at pH 7.4, 6.5, and 5.0 was 42%, 55%, and 75%, respectively (24 h). Biodistribution studies were performed. Resveratrol- and Dox-loaded micelles most effective in vivo.	Jia et al., 2019 [46]
Dextran-b-PLH	Doxorubicin	HuCC-T1 (+,−)	pH-dependent release of Dox from micelles at pH 6.8 compared to 7.4. Micelles had greater toxicity toward cells than free Dox as the pH was reduced to 7.0 and below.	Hwang et al., 2013 [53]
AAP-His	Paclitaxel	MCF7, S-180 (+,+)	Modest pH-dependent release of PTX (20% greater release of PTX at pH 5.0 than 7.4 (12 h)). pH-dependent cytotoxicity of MCF-7 cells observed. Marked in vivo inhibition of sarcoma-180 tumors with micelles.	Wang et al., 2017 [55]
mPEG-b-PLH/PEG-b-DSPE/2C5-PEG-b-DSPE	Paclitaxel	4T1 (+,−)	Mixed micelles were stable for several months at 4 °C. Micelles showed impressive pH responsiveness in releasing drug at pH 6.0 and 5.0. Micelles showed pH-dependent cytotoxicity, particularly with the 2C5 Ab, toward 4T1 cells.	Wu et al., 2013 [58]
PEG-Dox, PDPAEMA, H4R4	Doxorubicin	HeLa (+,−)	Low release of Dox at pH 7.4, yet significant release (90%) of the drug at pH of 5.5 (24 h). H4R4 had no role in drug release but likely enhanced endosomal lysis. NP had markedly improved efficacy toward HeLa cells vs. free drug.	Liang et al., 2014 [8]
**Branched Polymers**				
p(HEMA-co-His)-g-PLA, FA-PEG-PLA	Doxorubicin	HeLa (+,+)	pH-responsive release of Dox from micelles (10% release, pH 7.4; 65%, pH 5.0, 72 h). Folate-targeted mixed micelles had markedly greater inhibition of HeLa cells in vitro and HeLa xenografts in vivo than free Dox.	Tsai et al., 2010 [62]; Chen et al., 2021 [63]
(PIA-g-PEG-FA-g-PLH)	Doxorubicin	HeLa (+,−)	Very stable micelle at pH 7.4 that showed graded pH release of Dox at pH 7 and below. Greater than 90% of Dox was released at pH 5.0 (24 h). pH-dependent charge surface reversal. Folate-targeted micelle had greater toxicity for HeLa cells compared to free Dox.	Sun et al., 2015 [61]
PHEA-g-C18-His	Doxorubicin	HeLa (+,−)	Release of Dox showed modest pH dependence. Nearly 50% of Dox was not released at pH 5.0 (72 h). His-containing micelles showed more cytotoxicity than non-His micelles.	Yang et al., 2006 [64]
Star-shaped 5-armed PLGA-His	Docetaxel Disulfiram	MCF-7 (+,−)	Marked size increase in micelles at pH 6.8 vs. pH 7.4. Consistent with size increase, micelles released most of the two drugs at pH 6.8. Additionally, the pH-dependent micelles showed increased penetration into MCF-7 spheroids.	Swetha et al., 2021 [65]
HA-g-PLGA, mPEG-pHPMA	SN38	Tramp-C1 (+,+)	pH-sensitive release of imidazole polymer, mPEG-pHPMA, from micelle at pH 6.7. Additionally, coated micelles containing SN38 showed increased tumor targeting, deeper penetration, and enhanced efficacy against a prostate cancer tumor in vivo compared to noncoated vesicles.	Huang, W.-C. et al., 2016 [72]

^1^ Abbreviations. PEG-b-PLH, Polyethylene-glycol-b-poly-L-histidine; PEG-b-PLA, PEG-b-poly-L-lactide; FA-PEG-b-PLL, Folate-PEG-b-poly-L-lactide; PLA-PEG-PLH, poly-L-lactide-b-PEG-b-PLH; PEG-PLGA-PLH, PEG-b-poly(lactide-co-glycolide)-b-PLH; AAP-His, Histidine-modified auricularia auricula polymer; PEG-Dox, PEG-Doxorubicin conjugate; PDPAEMA, poly(2-(dipropylamino)ethyl methacrylate); H4R4, HHHH-RRRR; PIA-g-PEG-FA-g-PLH, poly(itaconic acid)-g-PEG-folate-g-PLH; PHEA-g-C18-His, poly(2-hydroxyethyl aspartamide)-g-C18-His; SN38, 7-ethyl-10-hydroxylcamptothecin; HA-g-PLGA, Hyaluronic acid-g-PLGA; mPEG-pHPMA, methoxy-PEGylated-b-p(histamine-methylacrylamide). ^2^ + or − indicate whether in vitro or in vivo experiments were done. ^3^ Cumulative release of drug at acidic pH—release of drug at 7.4.
